# Interatrial septal haematoma detected by intracardiac echocardiography during catheter ablation

**DOI:** 10.1093/ehjcr/ytae552

**Published:** 2024-10-15

**Authors:** Tsukasa Oshima, Kenichiro Yamagata, Katsuhito Fujiu

**Affiliations:** Department of Cardiovascular Medicine, The University of Tokyo Graduate School of Medicine, 7-3-1- Hongo, Bunkyo, Tokyo 113-8655, Japan; Department of Cardiovascular Medicine, The University of Tokyo Graduate School of Medicine, 7-3-1- Hongo, Bunkyo, Tokyo 113-8655, Japan; Department of Cardiovascular Medicine, The University of Tokyo Graduate School of Medicine, 7-3-1- Hongo, Bunkyo, Tokyo 113-8655, Japan

## Case description

**Figure 1:**
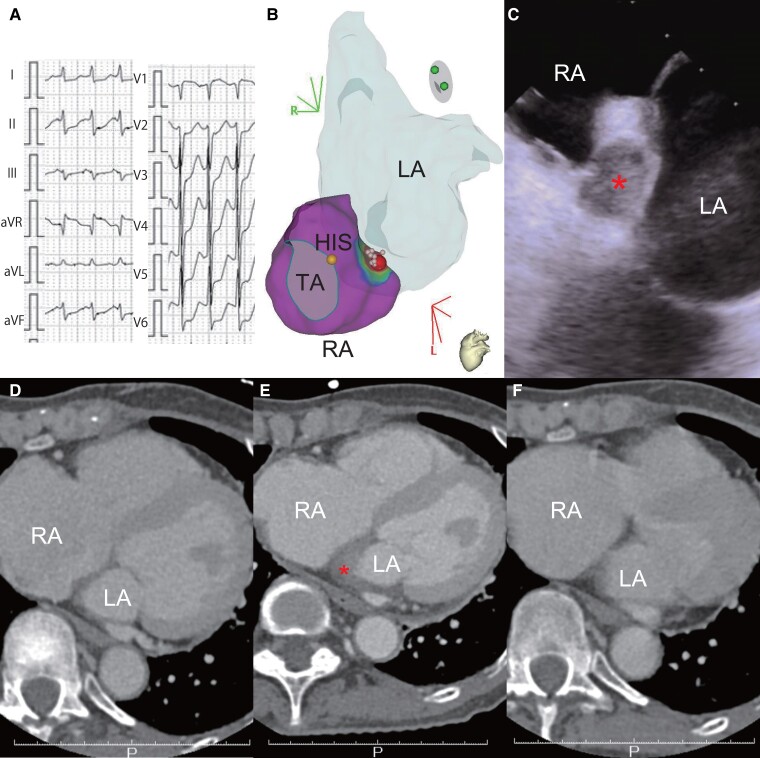
(*A*) Electrocardiogram of the atrial tachycardia. (*B*) Activation map of the three-dimensional mapping system revealed that the atrial tachycardia originated from the lower right atrium with an anatomical shell of the left atrium. Radiofrequency ablation was performed from the right atrium. A steam pop occurred at the red point. The pink points indicate other ablation sites. The yellow point represents the HIS, recording His bundle potential. RA, right atrium; LA, left atrium. (*C*). Intracardiac echocardiography revealed an echo-free space in the interatrial septum, indicating a haematoma (size 1.0 cm × 1.4 cm). The red asterisk indicates the echo-free space. (*D–F*). Computed tomography image at the level of the interatrial septum where the haematoma occurred. Although no haematoma was noted before the procedure (*D*), an interatrial septal haematoma was observed immediately after the procedure (*E*). It completely disappeared 3 months later (*F*). The red asterisk indicates the haematoma.

A 65-year-old female patient was admitted to our hospital with symptomatic paroxysmal atrial tachycardia (AT) that required catheter ablation. Activation mapping of both atria using the Octaray mapping catheter® (Biosense Webster, Irvine, CA, USA) revealed a focal AT pattern, with the earliest activation point at the lower right interatrial septum (IAS) (*Figure 1A* and *B*). The 8th application with ThermoCool SmartTouch^®^ (Biosense Webster) succeeded in suppressing the AT, and bonus applications were added around the successive site. Although the power setting was modest (30 W, contact force < 15 g, and <60 s), an audible steam pop occurred with a sudden increase in impedance. Thirty minutes later, intracardiac echocardiography (ICE) using Sound Star® (Biosense Webster) revealed an echo-free space in the IAS just around the ablation site, which was not observed before the pop (*Figure 1C*). Because this echo-free space occurred after the steam pop with no apparent blood flow connected to the right atrium, IAS haematoma was suspected. No epicardial effusion was detected, and blood pressure was continuously preserved. As the haematoma size measured using ICE remained unchanged and no further complications occurred, the procedure was completed 1 h later without AT inducibility, and she was returned to the normal ward with close monitoring of her vital signs. One week later, computed tomography revealed a haematoma that had not been identified prior to ablation (*Figure 1D* and *E*). This haematoma resolved spontaneously 3 months later without further complications (*Figure 1F*).

Interatrial septum haematoma is rare and may result in severe complications.^1^ We believe that early detection of IAS haematoma using real-time imaging with ICE is important to prepare for further deterioration. In the current case, it could have been difficult to detect the IAS without ICE as there were no indications of other symptoms, and observation with transthoracic echocardiography in this area is difficult.^2^ There are few reports of intramural haematomas. One study showed that intramural haematoma in the left ventricle required prompt surgical intervention.^3^ In our case, the haematoma was limited to the septal area, and careful observation with ICE allowed us to avoid surgical intervention. We speculate that the steam pop was due to repeated applications in a similar area and induced IAS rupture. However, surrounding structures, such as the annulus, limited the expansion of the haematoma, and the absence of a change in size suggested that the haematoma was benign.

Intracardiac echocardiography is useful for clarifying real-time structural information, which fluoroscopy and 3D mapping systems cannot provide. It also enables the early detection of complications before they become catastrophic.^3^ However, the benefit of ICE must be balanced against its additional cost.

##  

**Consent:** The patient provided informed consent for the publication of this report and associated images.

**Funding:** This research did not receive any specific grants from funding agencies in the public, commercial, or not-for-profit sectors.

## Data Availability

The data underlying this article are available in the article.
